# Phase II trial of standard versus increased transfusion volume in Ugandan children with acute severe anemia

**DOI:** 10.1186/1741-7015-12-67

**Published:** 2014-04-25

**Authors:** Peter Olupot-Olupot, Charles Engoru, Jennifer Thompson, Julius Nteziyaremye, Martin Chebet, Tonny Ssenyondo, Cornelius M Dambisya, Vicent Okuuny, Ronald Wokulira, Denis Amorut, Paul Ongodia, Ayub Mpoya, Thomas N Williams, Sophie Uyoga, Alex Macharia, Diana M Gibb, A Sarah Walker, Kathryn Maitland

**Affiliations:** 1 Department of Paediatrics, Mbale Regional Referral Hospital, Pallisa Road Zone, PO Box 921, Mbale, Uganda; 2 Department of Paediatrics, Soroti Regional Referral Hospital, PO Box 289, Soroti, Uganda; 3 Medical Research Council (MRC) Clinical Trials Unit, Aviation House, 125 Kingsway, London WC2B 6NH, UK; 4 Kilifi Clinical Trials Facility, KEMRI-Wellcome Trust Research Programme, PO Box 230, Kilifi, Kenya; 5 Wellcome Trust Centre for Clinical Tropical Medicine, Department of Paediatrics, Faculty of Medicine, St Marys Campus, Norfolk Place, Imperial College, London W2 1PG, UK

**Keywords:** Transfusion, Severe anemia, African children, Clinical trial, Infectious disease

## Abstract

**Background:**

Severe anemia (SA, hemoglobin <6 g/dl) is a leading cause of pediatric hospital admission in Africa, with significant in-hospital mortality. The underlying etiology is often infectious, but specific pathogens are rarely identified. Guidelines developed to encourage rational blood use recommend a standard volume of whole blood (20 ml/kg) for transfusion, but this is commonly associated with a frequent need for repeat transfusion and poor outcome. Evidence is lacking on what hemoglobin threshold criteria for intervention and volume are associated with the optimal survival outcomes.

**Methods:**

We evaluated the safety and efficacy of a higher volume of whole blood (30 ml/kg; Tx30: n = 78) against the standard volume (20 ml/kg; Tx20: n = 82) in Ugandan children (median age 36 months (interquartile range (IQR) 13 to 53)) for 24-hour anemia correction (hemoglobin >6 g/dl: primary outcome) and 28-day survival.

**Results:**

Median admission hemoglobin was 4.2 g/dl (IQR 3.1 to 4.9). Initial volume received followed the randomization strategy in 155 (97%) patients. By 24-hours, 70 (90%) children in the Tx30 arm had corrected SA compared to 61 (74%) in the Tx20 arm; cause-specific hazard ratio = 1.54 (95% confidence interval 1.09 to 2.18, *P* = 0.01). From admission to day 28 there was a greater hemoglobin increase from enrollment in Tx30 (global *P* <0.0001). Serious adverse events included one non-fatal allergic reaction and one death in the Tx30 arm. There were six deaths in the Tx20 arm (*P* = 0.12); three deaths were adjudicated as possibly related to transfusion, but none secondary to volume overload.

**Conclusion:**

A higher initial transfusion volume prescribed at hospital admission was safe and resulted in an accelerated hematological recovery in Ugandan children with SA. Future testing in a large, pragmatic clinical trial to establish the effect on short and longer-term survival is warranted.

**Trial registration:**

ClinicalTrials.Gov identifier: NCT01461590 registered 26 October 2011.

Please see related commentary article http://www.biomedcentral.com/1741-7015/12/68/abstract.

## Background

In sub-Saharan Africa severe anemia in children, defined as a hemoglobin less than either 5 g/dl or 6 g/dl, remains a leading cause of hospital admission [1] and a major factor in the 800,000 malaria deaths/year [2]. Young children and women account for more than three quarters of the blood transfusions in sub-Saharan Africa - most given as emergency life-saving treatments [3]. Despite high demand, blood supply is inadequate to meet the needs. On average only 2.3 units of blood are donated per 1,000 population in sub Saharan Africa, compared with 8.1 and 36.7 in medium and high-income countries [4]. Furthermore, most nationally funded blood services depend almost entirely on whole blood for transfusion, since the model of exclusive component preparation has technical, financial and possibly serious negative consequences and can only be feasibly implemented if substantially supported by external aid [3].

Owing to these resource-limitations, guidelines have been developed by the World Health Organization (WHO) that encourage the rational use of blood transfusion to treat severe anemia in order to prevent overuse and to reduce the risk of transfusion-transmitted infection [5,6]. Nevertheless, evidence is lacking regarding which hemoglobin threshold criteria, volume and timing of transfusion intervention are associated with optimal survival outcomes. Consequently adherence to the current guidelines is poor [7,8]. Children with severe anemia have high rates of in-hospital mortality (9% to 10%) [9], suggesting that the current recommendations are not optimal. An inadequate supply of blood to treat emergencies has previously been highlighted as a key factor driving poor outcomes, with 63% of the early deaths (<6 hours) occurring while awaiting transfusion [10]. A further consideration is whether the volume of transfusion is sufficient to correct the anemia. Currently, WHO recommends 20 ml/kg of whole blood (or 10 ml/kg packed cells) for all levels of anemia <4 g/dl, irrespective of initial hemoglobin or <6 g/dl if complicated by life-threatening features [5]. Few data are available on volumes received. One prospective study of pediatric transfusions in Siaya, Kenya, reported the mean volume of transfusion as 25 to 26 ml/kg whole blood [7]; however, 14% of transfusions were <15 ml/kg. Others following WHO guidelines (20 ml/kg) have shown only a modest hemoglobin rise of mean 2.5 to 3.3 g/dl [10-12] with approximately 25% remaining severely anemic (<5 g/dL) [10] following initial transfusion. Unpublished data from the Fluid Expansion as Supportive Therapy (FEAST) trial [13] indicates that of 1,422 children transfused, 322 (23%) received two or more transfusions, the proportion being greater (212/612, 35%) in those with hemoglobin <4 g/dl at enrollment. Multiple transfusions not only incur additional resource utilization but expose children to added risks of infection, transfusion reaction and adverse events. Using standard formulae to calculate the volume required [14], the current doses prescribed under-treat children with profound anemia by nearly 30% [15].

Larger initial transfusion volumes have not been systematically evaluated. We therefore investigated whether a greater initial volume of whole blood (30 ml/kg) compared to the standard recommendation (20 ml/kg whole blood) safely treated severe anemia with respect to a superior hematological correction and reduced the need for extra transfusions.

## Methods

### Design and treatment protocol

We conducted a multicenter open randomized Phase II trial, with the aim of providing preliminary results regarding the safety of higher volume transfusions and their feasibility and acceptability to clinicians and transfusion services. Eligible children from two clinical centers in Eastern Uganda (Mbale and Soroti Regional Referral Hospitals) were randomly assigned on admission to hospital (ratio 1:1) to receive either: (1) 20 ml/kg whole blood transfusion (Tx20) (alternatively 10 ml/kg of packed red blood cells (standard of care)); or (2) 30 ml/kg whole blood transfusion (Tx30) (or 15 ml/kg packed red blood cells). Sites were instructed to transfuse blood over three to four hours, as recommended by WHO.

### Study population

#### Screening procedure

**Figure 1:**
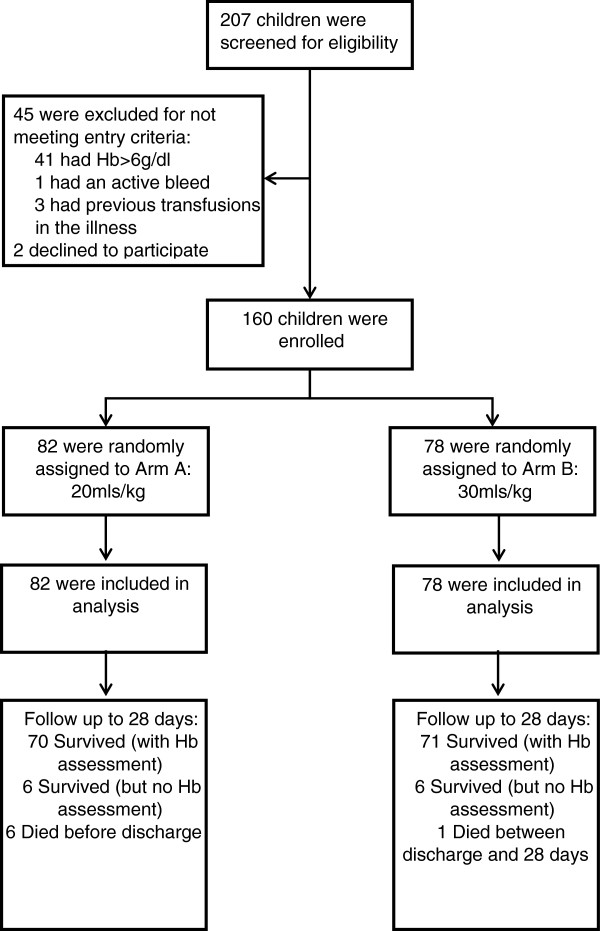
Trial flow.

We aimed to enroll 160 children, >60-days- and <12-years-old, with severe anemia at admission to the pediatric ward. Dedicated trial clinicians and nurses were employed to conduct the study. Potentially eligible children with clinical evidence of pallor were identified and registered in the eligibility screening log. A rapid bedside test by HemOcue (Ängelholm, Sweden) and bedside examination determined hemoglobin level and severity. Children were eligible if they had severe anemia (hemoglobin <6 g/dl) at the time of admission to hospital), no previous transfusion during the course of the current illness and a guardian or parent willing/able to provide consent (Figure 1: Trial Flow). Complicated severe anemia was defined as children with hemoglobin 4 to 6 g/dl in conjunction with markers of clinical severity (reduced conscious level or respiratory distress) or profound anemia (hemoglobin <4 g/dl). Children with malignancy, surgery, acute trauma or acute severe malnutrition were excluded from the study.

### Outcome measures

The primary outcome was correction of severe anemia (to hemoglobin >6 g/dl) at 24 hours. Secondary outcomes included: (1) meeting criteria for additional transfusion (development of profound anemia (hemoglobin <4 g/dl) or hemoglobin 4 to 6 g/dl with new markers of severity (impaired consciousness or respiratory distress)) from eight hours post randomization; (2) serious adverse events defined according to Good Clinical Practice [16] and including suspected pulmonary edema (bilateral basal crepitations with hypoxemia (oxygen saturations <90%); biventricular heart failure (severe tachycardia (<12-months-old: >180 beats per minute (bpm); 12-months to 5-years-old: >160 bpm; >5-years-old: >140 bpm) plus an increasing liver size) or suspected transfusion reaction; (3) mortality through 48 hours and 28 days post-admission; and (4) redevelopment of severe anemia (hemoglobin <6 g/dl) or mortality at 28 days post-admission.

### Study procedures

#### Consent

Prospective written, informed consent was obtained from parents or guardians of the children. The information sheet was in their usual language and was read aloud to those unable to read. Parents and guardians were encouraged to ask questions about the trial prior to signing the consent form. In cases where prior written consent from parents or guardians could not be obtained because of severity of the illness a provision was approved for verbal assent from a legal surrogate followed by delayed informed consent as soon as practicable.

The Mbale Research Ethics Committee, Mbale, Uganda approved the protocol. The trial was registered, prior to enrollment, with ClinicalTrials.Gov identifer: NCT01461590 (registered 26 October 2011).

### Randomization

Randomization was stratified by clinical center. The treatment allocation (Tx30 or Tx20) was kept in numbered, sealed opaque envelopes, each signed across the seal. The cards were numbered consecutively and opened in numerical order. The randomization list and envelopes were prepared before the trial by a statistician at the Kilifi Clinical Trials Facility and the list was not available to the investigators.

### Sample size

The study aimed to generate pilot safety and efficacy data on a higher transfusion volume (30 ml/kg) in children with severe anemia. Numbers required to address the trial objectives were therefore balanced against exposing children to a therapeutic intervention (dose) for which there are limited data. The overall sample size of 160 children (approximately 80 having signs of severity as defined above) randomized to 20 versus 30 ml/kg provided at least 80% power to detect major (20% to 25%) increases in the proportions experiencing the primary and secondary outcomes.

### Clinical monitoring and study assessments

Following consent and randomization, lactate (LactatePro®), glucose, malaria status (by blood film and Optimal® rapid diagnostic test (RDT)) and cross match were performed. Following national guidelines, HIV testing was conducted after completion of admission procedures, with pre- and post-test counseling done in accordance with routine practice. Blood was collected at admission into ethylenediaminetetraacetic acid (EDTA), stored at -80°C and typed by PCR for the hemoglobinopathies sickle cell anemia (HbSS), sickle cell trait (HbAS) and the common African variant of α-thalassemia and for the red cell enzymopathy G6PD deficiency at the end of the study, as described in detail previously [17], using DNA extracted using Qiagen DNA blood mini kits (Qiagen, Crawley, UK).

Transfusions were given in standard infusion sets incorporating a graded, filtered burette. Since the volume of the burette was only 150 ml, each transfusion, depending on volume, was given as consecutive aliquots (150 ml maximum/aliquot) until the transfusion was complete. For analysis, a separate transfusion was defined by a gap of 30 minutes or more between consecutive aliquots.

All children were reassessed at 30 minutes, 1 hour, 90 minutes, and 2, 4, 8, 16, 24 and 48 hours for consciousness level, vital signs (heart rate, oxygen saturation, respiratory rate, axillary temperature, blood pressure) and for adverse events. Hemoglobin was monitored 8-hourly on the day of admission and daily thereafter. Glucose and lactate were reassessed at 8, 16 and 24 hours, with lactate assessed again at 48 hours. At follow up (day 28) hemoglobin and malaria parasite status were reassessed.

Serious adverse event reports were sent to the Clinical Trials Facility, Kilifi, Kenya within two days and were also monitored against source documents by visiting monitors. An independent clinician removed all references to the randomized arm prior to review by the Endpoint Review Committee (ERC), which included an independent chair (JE), one independent clinician (IB), one clinician involved in trial management but not patient enrollment (KM) and one clinician not involved in the day-to-day running of the trial (DMG). The ERC had access to clinical narratives, bedside vital observations, serial laboratory and bedside blood tests and concomitant treatments. They adjudicated (blind to randomized arm) on whether fatal and non-fatal events could be related to transfusion or the volume transfused, and the main cause of death.

### Further management

An additional transfusion was permitted after eight hours (at the time of the first protocol hemoglobin reassessment) for children who still had either (1) hemoglobin <4 g/dl or (2) hemoglobin 4 to 6 g/dl and a sign of severity (respiratory distress or impaired consciousness). If a child required maintenance fluids, 5% dextrose was given at 3 to 4 ml/kg per hour until the child was able to drink. All children received standard treatments recommended by national guidelines, depending on their illness, including parenteral anti-malarials, antibiotics and/or antipyretics, anticonvulsants, oxygen (for oxygen saturations <90%) and glucose for hypoglycemia. Use of diuretics during blood transfusion was discouraged and reserved for children developing new signs suggestive of pulmonary edema or biventricular heart failure (defined as respiratory distress plus oxygen saturation <90%, bilateral basal crepitations, severe tachycardia and increasing liver size following transfusion).

### Statistical analysis

All analyses followed intention-to-treat and all statistical tests were two-sided. For the primary endpoint (correction of severe anemia at 24 hours), the arms were compared using Cox proportional hazards regression for the cause-specific hazard of a hemoglobin >6 g/dl before death, and the relative difference estimated by cause-specific hazard ratios. The cumulative incidence of severe anemia before death was also estimated; comparison of the sub-distribution hazard corresponding to the cumulative incidence between arms [18] gave similar results to the cause-specific hazards (not shown). Secondary outcomes were compared between arms using risk ratios and Fisher’s exact test. Other continuous characteristics (for example, volume and rates of the initial transfusion) were compared between arms using two sample t-tests assuming equal variance in the arms; categorical characteristics (for example, number of transfusions per child) were compared between arms using Fisher’s exact test. Change in vital signs, hemoglobin, glucose and lactate from baseline were compared between the arms using two sample t-tests at scheduled assessments assuming equal variance in the arms, and across all time points (global tests of difference) using generalized estimating equations (normal distribution, independent correlation structure).

## Results

### Study patients

Overall, 160 children were randomized between 10 October 2011 and 12 January 2012 (82 to Tx20, 78 to Tx30). All children included in the trial met eligibility criteria (Figure 1). Most baseline characteristics were broadly balanced between the arms (Table 1), although there were a few moderate differences as expected given the relatively small sample size. Median age was 36 months (interquartile range (IQR) 13 to 53). Signs of severity (respiratory distress or impaired consciousness) were present in 53% and 33% children, respectively. A total of 59% of the children had either a positive malaria slide and/or malaria RDT; median hemoglobin was 4.2 g/dl (IQR 3.1 to 4.9) with 46% having profound anemia (<4 g/dl); 31% had severe lactic acidosis (≥5 mmol/L). Twenty percent of participants had sickle cell anemia (HbSS), an observation consistent with this disease being a major risk factor for admission with severe anemia [19,20]. Conversely, sickle cell trait (HbAS), heterozygous and homozygous α-thalassemia and G6PD deficiency were found in lower frequencies than in the background population (unpublished observations), consistent with previous observations that these traits are associated with host protection from severe malaria [21-23].

**Table 1:** Baseline characteristics

	**Arm A: Tx20**	**Arm B: Tx30**	**Total**
	**20 ml/kg**	**30 ml/kg**	
**Variable**	**(Number = 82)**	**(Number = 78)**	**(Number = 160)**
**Demographic and anthropometric characteristics**			
Age months median (IQR)	36 (19 to 54)	31 (11 to 48)	36 (13 to 53)
Female sex – n/ total n (%)	41/82 (50)	40/77 (52)	81/159 (51)
Mid-upper arm Circumference ≤11.5 cm – n/total n (%)	1/80 (1)	3/78 (4)	4/158 (3)
Weight kg - median (IQR)	13 (9-16)	11 (8-15)	12 (9-15)
**Findings at presentation**			
Axillary temperature			
>37.5°C – n/total n (%)	39/81 (48)	37/78 (47)	76/159 (48)
<36°C (Hypothermia) – n/total n (%)	3/81 (4)	4/78 (5)	7/159 (4)
Oxygen saturation <90% - n/total n (%)	9/78 (12)	9/78 (12)	18/156 (12)
Moderate hypotension - n (%)	5 (6)	6 (8)	11 (7)
Dehydration - n (%)	6 (7)	6 (8)	12 (8)
Severe pallor (lips, gums, or inner eyelids) - n (%)	81 (99)	76 (97)	157 (98)
Consciousness level – n (%)			
Total n	81	78	159
Alert	51 (63)	56 (72)	107 (67)
Prostration	29 (36)	20 (26)	49 (31)
Coma	1 (1)	2 (3)	3 (2)
Convulsions during this illness - n (%)	6 (7)	13 (17)	19 (12)
Hemoglobinuria (dark urine) - n (%)	29 (35)	26 (33)	55 (34)
Jaundice visible to clinician - n (%)	43 (52)	44 (56)	87 (54)
Respiratory rate breaths/minute			
mean ± sd	47 ± 15	47 ± 13	47 ± 14
respiratory distress – n (%)	41 (50)	43 (55)	84 (53)
Heart rate beats/minute			
median (IQR)	153 (136 to 173)	156 (142 to 168)	155 (139 to 170)
Bradycardia (<80) - n (%)	1 (1)	0 (0)	1(1)
Severe tachycardia - n (%)^a^	30 (37)	26 (33)	56 (35)
Hepatomegaly – n/total n (%)	32/82 (39)	25/77 (32)	57/159 (36)
**Laboratory assessment**			
Hemoglobin - n (%)			
Median (IQR)	4.2 (3.0 to 4.8)	4.3 (3.3 to 4.9)	4.2 (3.1 to 4.9)
<4 g/dl	37 (45)	36 (46)	73 (46)
Glucose – n/ total n (%)			
<2.5 mmol/liter (45 mg/dl)	2/82 (2)	0/75 (0)	2/157 (1)
<3.0 mmol/liter (54 mg/dl)	2/82 (2)	0/75 (0)	2/157 (1)
Lactate ≥5 mmol/liter – n/total n (%)	23/82 (28)	26/76 (34)	49/158 (31)
Positive for HIV antibody - n/ total n (%)	1/39 (3)	0/38 (0)	1/77 (1)
Positive for malaria parasitemia - n (%)			
Negative on all tests done	33 (40)	32 (41)	65(41)
RDT positive, slide negative/unknown	13 (16)	15 (19)	28 (18)
RDT negative/unknown, slide positive	4 (5)	3 (4)	7 (4)
RDT positive, slide positive	32 (39)	28 (36)	60 (38)
**Genotypes**			
Sickle cell– n (%)			
AA (normal)	63 (77)	57 (73)	120 (75)
AS (sickle cell trait)	2 (2)	6 (8)	8 (5)
SS (sickle cell anemia)	17 (21)	15 (19)	32 (20)
Alpha thalassemia – n (%)			
Normal	45 (60)	48 (65)	93 (62)
Heterozygote	24 (32)	24 (32)	48 (32)
Homozygote	6 (8)	2 (3)	8 (5)
G6PD deficiency – n (%)			
Normal	56 (77)	55 (79)	111 (78)
Heterozygote/Hemizygote^b^	8 (11)	7 (10)	15 (10)
Homozygote	9 (12)	8 (11)	17 (12)

Note: denominators are all children randomized unless otherwise shown; ^a^defined as heart rate (HR) >180 for <1 year old, HR >160 for <5 years old, HR >140 for more than 5 years old; ^b^female heterozygote/male hemizygote. IQR, interquartile range; n, number; RDT, rapid diagnostic test; sd, standard deviation.

### Transfusions administered

All children received a transfusion and the initial volume actually infused followed the randomization strategy (within 5 ml/kg) in 80 (98%) patients in the Tx20 arm and in 75 (96%) in the Tx30 study arm. In the Tx20 arm, one child died before the transfusion was completed preventing them from receiving the full amount of blood, the other inadvertently received an initial transfusion of 30 ml/kg whole blood. In the Tx30 arm all three children not following the randomization strategy received less than 25 ml/kg in the initial transfusion, and two subsequently received additional transfusions. All initial transfusions were whole blood rather than packed cells. There was only one prescription of packed cells in the whole trial, given as a second transfusion (Tx30 arm). Median initial transfusion volumes were 20 ml/kg (IQR 20 to 20) and 30 ml/kg (IQR 30 to 30) in the respective arms (Table 2). Time to initial transfusion was similar between the two arms (*P* = 0.74), and was 98 minutes (IQR 75 to 128) overall, but transfusions were given signficantly faster in the Tx30 arm than in the Tx20 arm (median 7.6 versus 5.7 ml/kg/hour, respectively, *P* <0.0001). During the first 48 hours, 69 (88%) children assigned to Tx30 received only one transfusion; an additional eight and one patients received two or three transfusions, respectively. In Tx20, 67 (82%) children received only one transfusion and fifteen (18%) received two or more transfusions. These differences were not statistically significant (*P* = 0.23). Two patients in Tx20 and one patient in Tx30 received another transfusion after 48 hours.

**Table 2:** Volume, timing and additional transfusion by study arm

	**Arm A: 20 ml/kg (Number = 82)**	**Arm B: 30 ml/kg (Number = 78)**	***P *** **value**
Volume of initial transfusion^a^ (ml/kg) – median (IQR)	20 (20 to 20)	30 (30 to 30)	<0.0001
Rate of initial transfusion^a^ (ml/kg/hr) – median (IQR)	5.7 (4.9 to 6.7)	7.6 (6.1 to 8.4)	<0.0001
Time to initial transfusion (minutes) – median (IQR)	95 (75 to 128)	103 (75 to 130)	0.74
Total volume tranfused 0 to 48 hours (ml/kg) – median (IQR)	20 (20 to 20)	30 (30 to 30)	<0.0001
Number of transfusions^b^ per child 0 to 48 hours, number (%)			0.23
1	67 (82)	69 (88)	
2	14 (17)	8 (10)	
3	0 (0)	1 (1)	
4	1 (1)	0(0)	
Number of children with a transfusion after 48 hours	2	1	

^a^Refers to blood infused before the first 30-minute break between aliquots of blood. This was the entire initial prescription for all but three children (all 30 ml/kg arm) where the last aliquot was given 49, 64, and 92 minutes after the previous aliquot; ^b^refers to the number of prescriptions. IQR, interquartile range.

### Outcomes

**Figure 2:**
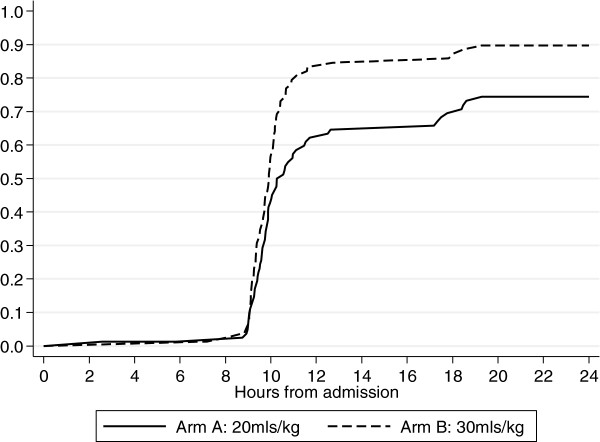
**Correction of severe anemia by 24 hours by study arm.** Time to first hemoglobin>6mg/dl by study arm by study arm (primary outcome- correction of severe anemia).

**Figure 3:**
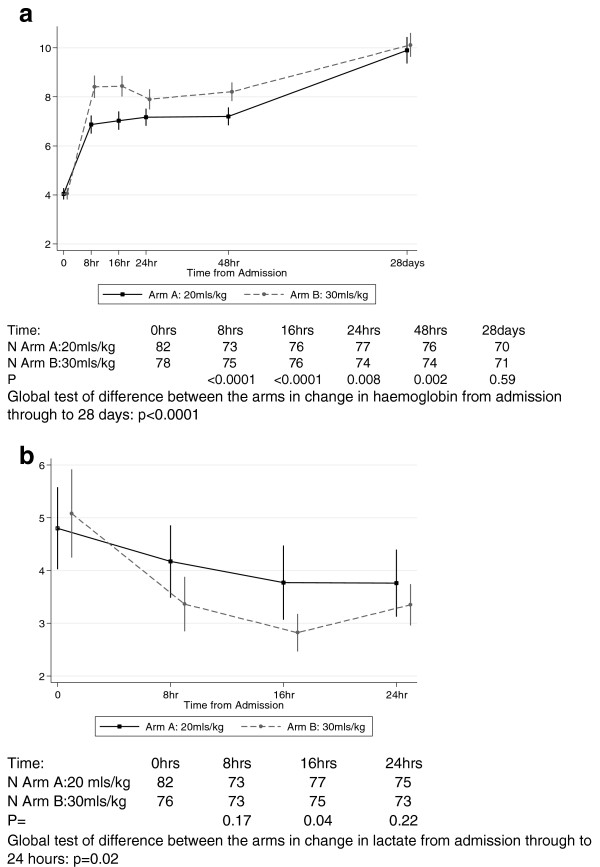
**Change in mean hemoglobin (3a) and lactate (3b) over follow up by study arm. a**. Hemoglobin over 28 days. **b**. Lactate levels over 24 hours.

By 24 hours after transfusion significantly more children in the Tx30 arm had corrected their severe anemia (primary endpoint) 70 (90%) versus 61 (74%) in the Tx20 arm; cause-specific hazard ratio for anemia correction before death = 1.54 (95% confidence interval (CI) 1.09 to 2.18, *P* = 0.01) (Table 3; Figure 2). There was also a trend towards more children in Tx20 than in Tx30 meeting the study criteria for additional transfusion (*P* = 0.06). Although more children in Tx20 had serious adverse events, differences were consistent with chance (*P* = 0.28, Table 3). By 28 days after transfusion, six children (7%) in Tx20 had died compared to one in Tx30 (*P* = 0.12; Table 3). There was also evidence for greater hemoglobin increases from enrollment in Tx30 versus Tx20 through to 28 days (global *P* <0.0001; Figure 3a) and faster reductions in lactate over the first 24 hours in Tx30 (global *P* = 0.02; Figure 3b). There was no evidence of differences in glucose between the two arms in the first 24 hours (global *P* = 0.09, Additional file 1: Figure S1). Differences in hemoglobin between the arms attenuated through follow-up, with the mean difference of 0.22 (95% CI -0.59 to 1.03) at day 28 not reaching statistical significance (*P* = 0.59). Although the combined endpoint of re-development of severe anemia or death at 28 days following admission also favored the arm receiving 30 ml/kg (4/72 (6%) compared to those in the standard of care (20 ml/kg) arm, 9/76 (12%)), the difference was not significant (*P* = 0.25). In total, eleven children did not attend the 28-day follow up; all (six Tx20, five Tx30) were traced in the community and survival status confirmed in ten (six Tx20, four Tx30). The last child (Tx30) was found to have died four days after discharge (included as a fatality below). There was no evidence of a difference between the arms in improvements in respiratory rate, heart rate or systolic blood pressure (global *P* >0.3). From admission to eight hours children in the Tx30 arm had a slightly (<2%) greater improvement in oxygen saturation: from eight hours oxygen saturation was similar in the two arms (global *P* = 0.003).

**Table 3:** Primary and secondary endpoints

**End point**	**Arm A: 20 ml/kg (N = 82)**	**Arm B: 30 ml/kg (N = 78)**	**Risk ratio (95% CI)**	***P *** **value**
Time to correction of severe anemia (by 24 hours) - number (%)	61 (74)	70 (90)	1.54^a^ (1.09,2.18)	0.01
Children meeting the criteria for additional transfusion - number (%)	12 (15)	4(5)	0.35 (0.12, 1.04)	0.06
SAE – number (%)	6 (7)	2 (3)	0.35 (0.07, 1.68)	0.28
Died before 48 hours – number (%)	4 (5)	0 (0)		0.12
Died before 28 days post-admission – number (%)	6 (7)	1 (1)	0.18 (0.02, 1.42)	0.12
Severe anemia or mortality at 28 days – number/total n^b^ (%)	9/76 (12)	4/72 (6)	0.47 (0.15, 1.46)	0.25

^a^cause specific hazard ratio for correction of severe anemia before death (results similar using competing risks sub-distribution hazard, not shown); ^b^denominator excludes children who were alive at 28 days but in whom hemoglobin was not measured. CI, confidence interval; SAE, serious adverse event.

### Serious adverse events

The independent ERC reviewed all serious adverse events (SAEs). There was one non-fatal SAE (transfusion reaction, Tx30) and seven fatal SAEs (Table 4). Six fatal events occurred in Tx20, all within hospital. Two of the fatalities had evidence of malaria infection. One fatal event occurred in Tx30, four days following discharge. None of the fatal events were judged by either the clinician or the ERC to be due to volume overload: in all fatal SAEs there was no indication of pulmonary edema, biventricular heart failure or transfusion-related acute lung injury. Fatal events were judged to be related to the severity of the underlying disease in three of six inpatient cases rather than to the transfusion, and possibly related to transfusion in three of six inpatient cases (all Tx20). The seventh fatality, which occured four days after discharge was judged unrelated to transfusion or transfusion volume. In fatal SAEs occurring in-hospital, transfusions occurred over 2.5 to 6.5 hours, and 6.8 hours in the child dying twelve days following admission and five days post-discharge from hospital (see Additional file 2: Table S1 for clinical details).

**Table 4:** Serious adverse events (including fatal events)

**SAE**	**Arm A: 20 ml/kg (Number = 82)**	**Arm B: 30 ml/kg (Number = 78)**	**Total**
Clinician defined SAEs			
Allergic reaction/transfusion reaction	0	1^a^	1
Deaths			
Cardio respiratory arrest	1	0	1
Respiratory arrest	1	0	1
Multiple organ failure	0	1^b^	1
Other deaths	4	0	4
**Total**	6	2	8
**Adjudication by endpoint committee**			
**Fatal events relationship to transfusion**^**c**^			
Definitely	0	-	
Probably	0	-	
Possibly	3	-	
Not related	3	1^b^	
unknown	0	-	

^a^Median rate of transfusion at the time of reaction, 0.95 ml/minute, no events judged to have any relationship to volume of transfusions; ^b^occurred four days after discharge (day 12 post-admission); ^c^see Table S1 in Additional file 2 for further details of the deaths.

## Discussion

We evaluated the safety and efficacy of a higher volume of initial transfusion (30 ml/kg) than currently recommended (20 ml/kg) in a controlled trial in 160 children presenting to two hospitals in Eastern Uganda with severe anemia with respect to hematological recovery, mortality, adverse events and the need for additional transfusion. More than half of the children had a febrile illness, 60% had evidence of current or recent *Plasmodium falciparum* malaria, and 50% and 30% had respiratory distress and/or severe lactic acidosis, respectively. Seven (4%) children died before 28 days following admission; six fatalities occurred prior to discharge. Children randomized to 30 ml/kg (Tx30) had a superior hemoglobin recovery at 24 hours (the primary outcome) and through to 28 days (global *P* <0.0001); the observed data suggest that the number of children meeting the criteria for repeated transfusion was lower (5% versus 15%, *P* = 0.06) and there was no indication that the higher initial volume resulted in an increase in adverse or fatal events compared to those in the Tx20 arm. The combined endpoint of re-development of severe anemia and survival at 28 days following admission also favored the arm receiving 30 ml/kg, but the fact that this was designed as a pilot safety trial meant that it was not powered to detect differences of this magnitude and did not reach statistical significance (*P* = 0.25). Of the eight adverse events adjudicated by the ERC, one was probably or definitely related to transfusion (non-fatal allergic reaction); the others were seven fatalities that either occurred in hospital and were unrelated (four) or possibly-related (three) to transfusion, or occurred four days after discharge.

This is the first trial to examine a higher initial volume of blood transfusion for treatment of severe life-threatening anemia in African children. Enrollment in the trial was pragmatic, with few exclusion criteria, at the point of admission to hospital. Most transfusions were started within 75 to 130 minutes of enrollment, indicating an efficient transfusion service, which may have underpinned the low aggregate in-hospital mortality (4%) that is much lower than published case-series and prospective studies in comparable study populations in Africa [10,11]. Children were managed in busy emergency rooms and pediatric wards. Children received an additional standard bundle of care, suggesting that implementation of such bundles, as well as urgent transfusion, could have also contributed to lower early mortality than in most of sub-Saharan Africa. Only one transfusion used packed cells rather than whole blood. The absence of adverse events related to volume overload provides reassuring endorsement of the relative safety of whole blood for pediatric transfusions in severely ill African children. Our study challenges the strong United States President’s Emergency Plan for AIDS Relief (PEPFAR) recommendation (made on the basis of patient safety) for component preparation, including packed cells [3]. It also supports the recent questions around implementation of these specific PEPFAR requirements for transfusion services, which are both costly and not evidence-based.

The trial was conducted at two hospitals in Eastern Uganda, in an area of intense all-year malaria transmission and where severe anemia as a cause of hospital admission is a major public health problem. Despite this, only 60% of the participants in the trial had evidence of malaria; and four of the six inpatient fatalities were in children with non-malarial febrile illnesses, indicating that the study has external validity for pragmatic management of children with severe anemia, including anemia with a non-malaria etiology. The study findings remain pertinent since, in many places, malaria has remained at the same or increased levels [24] and thus severe anemia remains a major cause of hospitalization in sub-Saharan Africa. The high frequency of children with sickle cell anemia has been noted previously in other hospital studies of severe anemia in malaria-endemic Africa [19]. The imbalance of the proportions with convulsion at admission most likely occurred due to chance (since the randomization was masked) and can occur in trials involving small numbers. Since convulsions are a risk factor for poor outcome we do not believe that these resulted in a lower mortality in the Tx30 arm [25]. Similarly, the higher in-hospital fatality rate in the 20 ml/kg transfusion arm (representing standard of care) was also consistent with chance, owing to the small size of the trial. Of note, virtually all transfusions in the trial were whole blood rather than packed cells, reflecting the difficulties local transfusion services have in preparing packed cells [3], and general lack of availability in populations similar to those studied here.

## Conclusions

This trial primarily demonstrated that a higher initial volume of transfusion (30 ml/kg) could be feasibly and safely implemented resulting in improvements in early hematological correction and global outcome, but also suggested a reduced need for repeat transfusion prescription compared to the usual standard of care (20 ml/kg) recommended by WHO guidelines. Since the WHO transfusion guidelines have not been systematically evaluated, this has resulted in variation in practice across African countries. Incomplete hematological response in children with severe anemia may underpin the poor outcomes including relapse, readmission [26] and death [9,26]. A policy to increase initial transfusion volume may also result in substantial cost savings, averting the overuse of scarce resources and decreasing the safety risk of further transfusion to the child; however, this cannot be recommended as the standard of care until tested in a larger trial. Further evaluation in a definitive randomized controlled trial examining efficacy and cost-effectiveness is therefore warranted.

### Key points

To address the poor outcomes of African children hospitalized with severe anemia we examined a higher initial transfusion volume than is currently recommended demonstrating its safety and a superior global outcome at 24 hours and 28 days after admission in Ugandan children with severe anemia.

## Competing interests

All authors declare they have no conflicts of interest.

## Authors’ contributions

The first draft was written by KM, together with ASW, DMG, and JT. CE, POO and JN are the lead site investigators in the trial and contributed to the drafting and revision of this manuscript. AM was responsible for study coordination and training and all other authors were responsible for patient enrollment and study conduct and contributed to the intellectual content and revision of this manuscript. PO and DA were responsible for coordination of data collection and quality control. MC, TS, CMD, VO, RW participated in the data collection and interpretation of data. TNW, SU and AM were responsible for the DNA extraction and genotyping and interpretation of data. All authors read and approved the final manuscript. Kathryn Maitland is the guarantor of the article.

## Pre-publication history

The pre-publication history for this paper can be accessed here:

http://www.biomedcentral.com/1741-7015/12/67/prepub

## Supplementary Material

Additional file 1: Figure S1 Glucose level over 24 hours. **Figure S2.** Heart rate over 48 hours. **Figure S3.** Respiratory rate over 48 hours. **Figure S4.** Systolic blood pressure over 48 hours. **Figure S5.** Oxygen saturation over 48 hours. Click here for file

Additional file 2: Table S1 Adjudication of serious adverse events and deaths by endpoint review committee: clinical narratives. **Table S2.** Assignment of causality. Click here for file
