# Supplementary material for: Phase II trial of standard versus increased transfusion volume in Ugandan children with acute severe anemia
Source: BMC Med. 2014 Apr 25;12:67. doi: 10.1186/1741-7015-12-67 (PMC4101869; doi:10.1186/1741-7015-12-67)
Supplement: Additional file 1: Figure S1 — Glucose level over 24 hours. Figure S2. Heart rate over 48 hours. Figure S3. Respiratory rate over 48 hours. Figure S4. Systolic blood pressure over 48 hours. Figure S5. Oxygen saturation over 48 hours. [file 1741-7015-12-67-S1.doc]

**Supplementary Figure 1: Glucose level over 24 hours**

| Time: | 0hrs | 8hrs | 16hrs | 24hrs |
| --- | --- | --- | --- | --- |
| N Arm A: 20mls/kg | 82 | 78 | 75 | 77 |
| N Arm B: 30mls/kg | 75 | 78 | 78 | 77 |
| P |  | 0.83 | 0.42 | 0.92 |

Global test of difference between the arms in change in haemoglobin from enrolment through to 24 hours: p=0.09

**Supplementary figure 2: Heart Rate (beats/min) over 48 hours**

| Time: | 0hrs | 2hrs | 4hrs | 8hrs | 16hrs | 24hrs | 48hrs |
| --- | --- | --- | --- | --- | --- | --- | --- |
| N Arm A: 20ml/kg | 82 | 70 | 81 | 76 | 77 | 77 | 77 |
| N Arm B: 30 ml/kg | 78 | 68 | 78 | 78 | 77 | 77 | 76 |
| P |  | 0.07 | 0.57 | 0.24 | 0.11 | 0.71 | 0.91 |
| Global test from enrolment to 48 hours p=0.32 | | | | | | | |

**Supplementary figure 3: Respiratory rate (breaths/min) over 48 hours**

| Time: | 0hrs | 2hrs | 4hrs | 8hrs | 16hrs | 24hrs | 48hrs |
| --- | --- | --- | --- | --- | --- | --- | --- |
| N Arm A: 20ml/kg | 82 | 70 | 80 | 76 | 77 | 77 | 77 |
| N Arm B: 30 ml/kg | 78 | 68 | 78 | 78 | 78 | 77 | 76 |
| P |  | 0.93 | 0.85 | 0.68 | 0.54 | 0.83 | 0.95 |
| Global test from enrolment to 48 hours p =0.76 | | | | | | | |

**Supplementary figure 4: Systolic Blood Pressure (mmHg) over 48 hours**

| Time: | 0hrs | 2hrs | 4hrs | 8hrs | 16hrs | 24hrs | 48hrs |
| --- | --- | --- | --- | --- | --- | --- | --- |
| N Arm A: 20ml/kg | 79 | 70 | 81 | 76 | 77 | 77 | 77 |
| N Arm B: 30 ml/kg | 77 | 68 | 78 | 78 | 77 | 77 | 76 |
| P |  | 0.26 | 0.41 | 0.28 | 0.24 | 0.44 | 0.23 |
| Global test from enrolment to 48 hours p=1.00 | | | | | | | |

**Supplementary figure 5: Oxygen Saturation (%) over 48 hours**

| Time: | 0hrs | 2hrs | 4hrs | 8hrs | 16hrs | 24hrs | 48hrs |
| --- | --- | --- | --- | --- | --- | --- | --- |
| N Arm A: 20mls/kg | 78 | 67 | 78 | 74 | 77 | 77 | 77 |
| N Arm B: 30 mls/kg | 78 | 67 | 78 | 78 | 76 | 76 | 76 |
| P |  | 0.13 | 0.50 | 0.94 | 0.42 | 0.37 | 0.48 |
| Global test from enrolment to 48 hours p= 0.003 | | | | | | | |
