# Supplementary material for: Phase II trial of standard versus increased transfusion volume in Ugandan children with acute severe anemia
Source: BMC Med. 2014 Apr 25;12:67. doi: 10.1186/1741-7015-12-67 (PMC4101869; doi:10.1186/1741-7015-12-67)
Supplement: Additional file 2: Table S1 — Adjudication of serious adverse events and deaths by endpoint review committee: clinical narratives. Table S2. Assignment of causality. [file 1741-7015-12-67-S2.doc]

**Table S1: Adjudication of serious adverse events and deaths by endpoint review committee: clinical narratives**

| Endpoint Review Committee (ERC), 4 members (one chair) reviewed clinical narratives, bedside vital observations, serial laboratory and bedside blood tests and concomitant treatments. Details of randomized transfusion arm had been removed (black ink or black highlight on PDF versions).  **Adjudication** Whether there was a causative relationship between the reported event and the trial intervention (see Table S2 for Causality terminology)  **Question 1** Whether fatal and non-fatal events could be related to transfusion?  **Question 2** Was there a causative relationship between the reported event and the trial arm (volume)?  **Additional comments** on main cause of death and possible diagnosis | | | |
| --- | --- | --- | --- |
|  | **Consensus:** meeting 9^th^ March 2013 without knowledge of randomised arm | **Clinical details** | **Actual Randomized Arm** |
| **MTx013**  Fatal | **Unrelated**  1/ Not transfusion related  2/ Not related to volume received  Fatality related to severity of illness: severe malarial anaemia; acidosis; hypoxia and recurrent hypoglycaemia  Multiple organ failure | **Clinical History and admission findings**  A 2 year old admitted with history of fever, cough, vomiting and lethargy. Past history of repeated blood transfusions.  **Vitals at admission** Comatose, with severe tachycardia (heart rate (HR) 189 beats/min) severe respiratory distress (respiratory rate (RR) 62 breaths/min), Kussmaul’s breathing and hypoxia (oxygen staturation 86% on air)  **Examination** Severe pallor, chest auscultation: clear no crackles, no evidence of heart failure. Hepatomegaly 2 cm below costal margin (BCM) and splenomegaly of 2 cm BCM.  **Admission investigations** positive malaria rapid diagnostic test (RDT), Haemoglobin (Hb) 3.0g/dL, lactate 15.1mmols/L and blood glucose of 1.9mmols/L.  **Treatment**  Oxygen, glucose and started second-line intravenous antibiotics. Transfusion was ordered but started 90 mins post-admission.  **Clinical progress**   - 30 minutes after starting the transfusion, he remained in coma, temperature of 38.4^0^C, HR 121 beats/min, RR 37 breaths/min. O_2_ sats 67% on face mask oxygen. On chest auscultation - no evidence of crepitations. - One hour of starting transfusion: comatose, evidence of impaired perfusion (capillary refilling time (CRT) 3 secs, HR 151 bpm, SBP 77mmHg). RR 30 breaths/min, O_2_ sats unrecordable on pulse oximetry. Attending doctor noted coarse crepitation and mouth secretions and queried aspiration. - Hypoglycaemia was noted (2.6 mmols/L) and corrected with intravenous dextrose. The child died shortly after this.   **Attending clinicians diagnosis**: Death to be due to respiratory arrest and multiple organ failure as a consequence of lactic acidosis and hypoxia caused solely by the severity of the disease process. | **20mls/kg** |
|  |  |  |  |
| **STx027**  Fatal | **Possible**  1/ Possibly transfusion-related  2/ Possibly volume and transfusion rate  Cannot rule out speed of transfusion being related  Sickle cell suspected  Other | **Clinical History and admission findings**  A 4y 6m old who was admitted with complaints of high-grade fever on and off associated with bone pains.  **Vitals** Alert, temperature 36.8^0^C, HR 114 beats/min, RR 48 breaths/min, SBP 114mmHg O_2_ sats 97% on air  **Examination:** pale, jaundices and had obvious musculoskeletal pain  **Admission investigations** showed a negative malaria RDT, Hb3.9g/dL, blood sugar 7.7mmols/L, HIV (-ve), lactate 4.2mmols/L  **Treatment:** First-line intravenous antibiotics and transfusion started  **Clinical progress**   - Review at 30 minutes stable. - One hour: Rise in temperature and heart rate. However the respiratory state remained the same. Blood transfusion was stopped and tepid sponging done then transfusion was re-started. - Vitals done at 4 hours: conscious level had dropped to **V** from **A** on the AVPU scale. Temperature 38.1^0^C, HR 160 bpm RR 42 breaths/min O_2_ sats 92% on air. CRT 3 secs and SBP was 100mmHg. Blood transfusion was still running at this time. - 4hours 45 minutes (transfusion ongoing), patient was found to be hypoxic O_2_ sats 88% on air and oxygen by face mask started. Temperature 37.5^0^C, HR 101 bpm; RR 28 breaths/min CRT 3 secs and SBP 71mmHg. The child conscious level had decreased (**P** on the AVPU scale). Blood glucose was 17.3mmols/L with lactate of 13mmols/L. - Soon after, patient started gasping and had oral secretions oozing that required suctioning. Blood transfusion was stopped once again and resuscitation was started. Despite resuscitation, patient succumbed.   **Attending clinicians diagnosis**: Death was believed to have been caused by the severity of the underlying disease process. | **20mls/kg** |
|  |  |  |  |
| **STx053**  Fatal | **Possible**  1/ Possibly related to transfusion  2/ Possibly related to volume/rate  But more likely to underlying illness  Pulmonary oedema  cannot be ruled out.  Respiratory | **Clinical History and admission findings**  A 3y 7m admitted with 2 day history of fever and an episode of passing dark coloured urine. This was 9th admission with 7 previous transfusions.  **Vitals** Prostration, hypothermia (35.2^0^C), HR 131 beats/min, RR 40 breaths/min; O_2_ sats 86% on air. SBP was 98mmHg.  **Examination** severe pallor, clinical jaundice and labored breathing, lung fields clear on auscultation.  **Admission investigations** positive malaria RDT/slide, Hb 4.7g/dL, blood glucose 1.4mmols/L, lactate 14.6mmols/L HIV (-ve).  **Treatment** Glucose was corrected intravenously and also started on Artemether and first line antibiotics.  **Clinical Reviews**   - 30 minutes into transfusion, the conscious level remained depressed and temperature was 34.5^0^c, heart rate 161 beats/min, respiratory rate 45 breaths/min with unrecordable oxygen sats. Evidence of compensated shock (CRT=3 secs, temperature gradient and SBP was 110mmHg). The oxygen sats remained unrecordable throughout as the child had a persistent temperature gradient with CRT of 3 secs and tachycardia. - At 1 hour, hypoglycemia was persistent, (1.8mmols/L) and was again corrected. In the course of transfusion, he passed dark coloured urine twice and continued to have persistent hypoglycemia and hypoxia. - 4 hours into the transfusion, breathlessness worsened and he was found to have excessive oral secretions. He suffered an arrest and succumbed despite resuscitation.   **Attending clinician diagnosis** Death to have been due to possible shock and multiple organ failure. | **20mls/kg** |
|  |  |  |  |
| **STx051**  **Fatal** | **Possible**  1/ Possible related to transfusion  2/ not related to volume  Rapid decline in consciousness and hypoxia (clear lung fields) suggested central nervous system event  ?partial seizure or seizure related to hypoglycaemia  Multiple organ failure | **Clinical History and admission findings**  A 10 month old admitted with history of fever and cough but no difficulty in breathing.  **Vitals** Alert, temperature 38.3^0^C, HR 132 bpm, RR 52 breaths/minO_2_ sats 90% on air and SBP 77mmHg.  **Admission investigations** negative malaria RDT/malaria slide, Hb 5.5g/dL, glucose 8.3mmols/L, lactate 2.8mmols/L, HIV (-ve)  **Treatment**  Started on first line antibiotics and oxygen and transfusion started.  **Clinical Reviews**   - After 30 minutes of transfusion, conscious level had dropped to **P** on the AVPU scale. Temperature was 38.9^0^C, HR 198 bpm, RR 64 breaths/min hypoxic (O_2_ sats 76% on oxygen by face mask) and SBP 86mmHg - Patient had persistently high temperatures throughout transfusion despite tepid sponging and being on paracetamol. Heart rate and respiratory rate rose from the time transfusion was started and remained persistently high throughout. O_2_ sats fluctuated between 70% and 80% most of the time. - About 6 hours into admission, the child suffered what appeared to be respiratory arrest and attempts at resuscitation proved unsuccessful.   **Attending clinician diagnosis** death to have been related to both the complications of the transfusion and also as a consequence of severe anaemia. | **20mls/kg** |
|  |  |  |  |
| **MTx067**  **Fatal** | **Unrelated**  1/ to transfusion  2/ to volume of transfusion  Child developed an acute abdomen  ? perforation? sepsis  ? Diclofenac caused acute bleed  Cardiovascular collapse | **Clinical History and admission findings**  A 10y 10m old who was admitted with history of fever, cough and difficulty in breathing for 9 days. She had been unwell for 2 months on and off and had been seen and treated at peripheral facilities a number of times.  **Vitals** Alert, Temperature 39.2^0^C, HR 144 beats/min, RR 44 breaths/min, O_2_ sats 97% on air. **Examination** respiratory distress lung fields clear on ausculation, hepatomegaly (4 cm BCM) and splenomegaly 7cm.  **Admission investigations** negative malaria RDT, Hb 3.9g/dL, glucose 8.4mmols/L and lactate 3.2mmols/L.  **Treatment** After admission patient was started on first line antibiotics and diclofenac (2 doses) together with blood transfusion.  **Clinical Progress**  She remained stable throughout transfusion.  At 8 hours Hb had risen to 6.7g/dL and condition remained stable but fevers were persistent.  **Day 3:** repeat RDT for malaria was positive. Commenced on intravenous quinine resulting in steady improvement.  **Day 5** Sudden deterioration in clinical condition: conscious level dropped and child very restless . Hb 2.6g/d and blood glucose unrecordably low. After correction of hypoglycemia, a second transfusion was started.  Post transfusion Hb 5.8g/dL, however 5 hours later, she again became very restless, was sweating and had cold extremities. Attending clinician examined the abdomen and it was quite tender generally and had features of peritonitis.  Vitals: Un-recordable blood pressure and severe respiratory distress although chest auscultation found lung fields were clear. Severe hypoglycaemia: glucose 1.3mmols/L (for which she received a glucose correction) and severe metabolic acidosis (Lactate 16.8mmols/L). Soon after she suffered respiratory arrest and succumbed despite resuscitation.  **Attending clinician diagnosis** death to have been due to cardiovascular collapse suspected peritoneal perforation. | **20mls/kg** |
|  |  |  |  |
| **MTX014**  **Late**  **Fatality**  **(Day 12)** | **Unrelated**  1/ to transfusion  2/ to volume of transfusion  Possible diagnosis  Hypersplenism and unresolved sepsis  Other | **Clinical History and admission findings**  8 years boy admitted with 1 week of fever, cough and vomiting.  **Vitals:** Alert. Temperature 37.5^0^c, Heart rate 139 bpm, RR 45 breaths/min and O_2_ sats 93% on air. SBP 83mmHg.  **Examinatio**n: Severe pallor, respiratory distress (indrawing and deep breathing). Hepatomegaly and splenomegaly of 12cm below the costal margin.  **Admission investigations** negative malaria RDT, Hb 2.5g/dL, Blood sugar 3.1mmols/L and lactate 5.1mmols/L. HIV test was not done.  A diagnosis of severe anaemia with hypersplenism secondary to septicaemia was made by the attending clinician.  **Treatment** After admission patient was started on first line antibiotics and given oxygen, and blood transfusion started.  **Clinical Progress**  Monitoring throughout transfusion showed that the child remained generally stable and at  **8 hours** Hb 5.2g/dL, lactate 8.9mmols/L and Blood sugar 11.9mmols/L. Vitals Temperature 37.2^0^C, HR 112 beats/min, RR 56 breaths/min and O_2_ sats 97% and SBP 80mmHg.  Child remained relatively stable but about 21 hours after admission, the attending clinician decided to give a repeat transfusion on the basis of a clinical picture of respiratory distress. The child received a second transfusion. The second transfusion ended uneventfully.  **48-hour review** Hb =6.8d/L; child was stable but spiking fevers. Gradually improved over next few days and discharged on the 7^th^ day of admission. At discharge he was ambulant and able to feed himself.  **Day 28 review**: child defaulted and the team contacted his parents who reported he died about 5 days after discharge. The child had fallen ill with similar complaints 4 days after discharge and was taken to hospital but died soon after arrival in hospital. The parents were informed the child required blood transfusion but he died before transfusion was initiated. Details of the clinical picture in the period leading to death are unavailable. | **30mls/kg** |
|  |  |  |  |
| **STX055**  **Non-fatal; allergic reaction** | **Probably**  1/ related to transfusion  2/ not related to volume | **Clinical History and admission findings**  A 1y 9m old female child admitted with fever for 7 days. The child had received some unknown medication from a peripheral facility before referral to the hospital.  **Vitals:** alert, afebrile, HR 152 beats/min, CRT > 3 secs, no respiratory distress and O_2_ sats of 98% on air. Examination severe pallor but no localizing sigsn  **Admission investigations** positive RDT for malaria, Hb 4.5g/dL,glucose 7.1mmols/L, lactate 2.9mmols/L.  **Treatment** Child was started on blood transfusion together with first line antibiotics and quinine.  **Clinical Progress**  After about 100mls of whole blood had been transfused, the child developed generalized body itching and urticarial rash around the face. The transfusion was stopped, hydrocortisone was given intravenously. Blood was re-started and after further 50mls of blood transfused the rash and itchiness worsened and so the transfusion was stopped. Group and cross matching were re-checked - no errors were reported.  About 2 hours following the discontinuation of the transfusion the pruritus and urticarial rash were noted to be subsiding. So transfusion completed with a new (different) batch of blood without any adverse reaction.  8 hours review: Hb 8.4g/dL lactate 5.9mmols/L. Thereafter, continued inpatient care was uneventful. On both day 7 and day 28 reviews, child was also found to be stable with no residual complaints. | **30mls/kg** |

**Supplementary Table 2:** Assignment of causality

| **Relationship** | **Description** |
| --- | --- |
| **Unrelated** | There is no evidence of any causal relationship |
| **Unlikely** | There is little evidence to suggest there is a causal relationship (e.g. the event did not occur within a reasonable time after administration of the trial medication). There is another reasonable explanation for the event (e.g. the participant’s clinical condition, other concomitant treatment). |
| **Possible** | There is some evidence to suggest a causal relationship (e.g. because the event occurs within a reasonable time after administration of the trial medication). However, the influence of other factors may have contributed to the event (e.g. the participant’s clinical condition, other concomitant treatments). |
| **Probable** | There is evidence to suggest a causal relationship and the influence of other factors is unlikely. |
| **Definitely** | There is clear evidence to suggest a causal relationship and other possible contributing factors can be ruled out. |
| **Not assessable** | There is insufficient or incomplete evidence to make a clinical judgement of the causal relationship. |
